# Sexual abuse survivors in Ankara, Turkey: Understanding the impact of post-traumatic stress disorder on self-injury

**DOI:** 10.5339/qmj.2024.69

**Published:** 2024-12-20

**Authors:** Helin Aburşu, Selma Tural Hesapçioğlu, Mehmet Fatih Ceylan

**Affiliations:** ^1^Faculty of Medicine, Yildirim Beyazit University, Ankara, Turkey *Correspondence: Ovais Wadoo. Email: helinabursu@gmail.com

**Keywords:** Self-injury behaviors, post-traumatic stress disorder, major depressive disorder, sexual abuse, suicide, Turkey

## Abstract

**Introduction:** Self-injury (SI) behavior represents a significant mental health concern that is prevalent among children and adolescents. The aim of this study was to examine the rates and types of suicidal and non-suicidal SI among post-traumatic stress disorder (PTSD) cases of sexual abuse victims and compare them with a major depressive disorder (MDD) group and a healthy control group.

**Methods:** This retrospective study focused on patients seeking treatment in the Ankara Yildirim Beyazit University Yenimahalle Education and Research Hospital Child and Adolescent Psychiatry Department between 2018 and 2021 in Ankara, Turkey. The study included patients diagnosed with PTSD and MDD following sexual abuse in the PTSD group and assessed standardized scales such as the child depression inventory, screen for child anxiety related emotional disorders, and clinical global impression.

**Results:** The study included 46 cases in the PTSD–MDD group, 60 in the MDD group, and 47 in the control group. The PTSD–MDD group had significantly higher levels of SI (*p* < 0.05). The predominant form of SI in this group was self-cutting. Moreover, those with sexual abuse were four times more likely to attempt suicide (OR = 4.1), and the non-suicidal self-injury (NSSI) group was 12 times more likely to attempt suicide (OR = 12.7).

**Conclusion:** These findings highlight the increased risk of self-harm and suicidal behavior in individuals diagnosed with PTSD–MDD who have been sexually abused, particularly highlighting the significant impact of NSSI and its association with increased suicide risk.

## INTRODUCTION

Self-injury behavior is a mental health problem affecting 17–18% of adolescents worldwide.^1^ There are two types of self-injury behavior: suicidal self-injury (SSI) and non-suicidal self-injury (NSSI). The main criterion for distinguishing NSSI behavior from suicide is the intention to die.^2^

Suicide is a critical problem and one of the leading causes of death among adolescents worldwide.^3^ Many factors affect suicide attempts and suicidal thoughts. Some of these factors include child abuse, neglect, domestic violence, poverty, and family psychopathology.^4–6^

Research shows that NSSI is also a predictor of SSI behavior.^7,8^ NSSI can be of different types, such as cutting, hitting, scratching, burning, hair pulling, pinching, and biting. The highest rate of self-injury is self-cutting^9,10^. Self-injury behaviors can have several intentions: emotion regulation, desire to influence others, self-punishment, coping with difficulties, and emotion-seeking.^11^

There is a connection between childhood trauma and suicidal behaviors. Neglect, emotional, and physical abuse, especially sexual abuse in childhood, increase the risk of NSSI and SSI behavior.^12^ Furthermore, in some cases, childhood sexual abuse may contribute to the development of self-harming behavior through mediating variables such as depression, anxiety, and self-derogation, each of which is known to be associated with both childhood sexual abuse and self-harming behavior.^12^ Many studies have shown that childhood sexual abuse is associated with self-injury behavior also in adulthood.^13,14^

SSI and NSSI occur in many psychopathologies. Some of these include major depressive disorder (MDD), post-traumatic stress disorder (PTSD), oppositional defiant disorder, and conduct disorder.^15^ Nearly half of the cases diagnosed with PTSD are also diagnosed with MDD.^16,17^ In some hypotheses, the PTSD–MDD comorbidity is also called post-traumatic mood disorder^18^. Some studies in adults showed that PTSD–MDD comorbidity was more related to SSI and NSSI behaviors than to MDD alone.^19,20^ Studies investigating the relationship between sexual abuse and the diagnosis of PTSD in children and adolescents with NSSI and self-injury are lacking in the literature. Studies found in the literature predominantly focus on the implications of childhood sexual abuse into adulthood.^19,20^

In Turkey, to facilitate the least traumatizing process for children who are victims of sexual abuse, a comprehensive evaluation is conducted by an experienced forensic interviewer in a forensic interview room. This process is coordinated through joint efforts of the Ministry of Health and other relevant ministries. Children's statements are obtained in the presence of experts, except in compelling circumstances, to mitigate potential secondary victimization. At the discretion of public prosecutors, forensic psychiatric and physical examinations are also conducted to initiate the judicial prosecution process.

In this retrospective study, self-injurious behaviors among pediatric and adolescent patients diagnosed with PTSD–MDD were carefully compared with those diagnosed with MDD alone. A control group was also included for comparison. The primary focus was on understanding how PTSD and sexual abuse influenced these behaviors.

## METHODS

### Participants

The study analyzed the records of 1,439 patients who attended the Child and Adolescent Psychiatry Outpatient Clinic at Yildirim Beyazit University Yenimahalle Education and Research Hospital in Ankara, Turkey, from 2018 to 2021. Following a retrospective review, 153 patients were included in the study based on specific inclusion criteria. Diagnoses were determined using the Diagnostic and Statistical Manual of Mental Disorders, Fifth Edition (DSM-5) diagnostic system. The study included 46 participants in the PTSD–MDD group, 60 in the MDD group, and 47 children and adolescents in the control group. Inclusion criteria for the PTSD–MDD group were exposure to sexual abuse and comorbid diagnoses of PTSD and MDD related to the abuse. Participants in the MDD group were included based on a diagnosis of MDD without a history of sexual abuse or PTSD. The control group included children and adolescents who sought counseling at the clinic, had no history of sexual abuse, and had no psychiatric disorders after comprehensive evaluations. All participants were between 6 and 18 years old, and those diagnosed with psychotic disorders or clinically diagnosed with intellectual disabilities were excluded from the study. The study was approved by the Ethics Committee of Yenimahalle Education and Training Hospital on August 18, 2021, with IRB number E-2021-36.

### Data collection tools

#### Child depression ınventory

The child depression inventory (CDI), developed by Kovacs, is a self-report scale completed by children aged 6–18 years.^21^ It consists of 27 items, each of which offers three response options. A higher score indicates a more severe level of depression, with a maximum score of 54 and a cutoff point set at 19. Öy et al. conducted a Turkish validity and reliability study for the scale.^22^

#### Screen for childhood anxiety and related emotional disorders

Karaceylan Çakmakçı conducted the Turkish validity and reliability study for the scale originally developed by Birmaher et al.^23,24^ During the assessment, the child is asked to select the most suitable option for each statement. This can be completed either by the child reading it independently or by reading it to them. A higher score indicates a higher general level of anxiety.^23,24^

#### Clinical global impression–severity of illness

The clinical global impression–severity of illness (CGI-SI) was developed by the American National Mental Health Institute and published in 1976.^25^ The CGI scale consists of three questions including severity of disease, recovery, and severity of side effect. Only the severity index section was used in this study. CGI-SI is a Likert-type scale with a total of seven values. A person with a psychiatric disorder is evaluated between 1 and 7 points depending on the severity of the disorder at the time of completing the scale: 1 = normal, not at all ill; 2 = borderline mentally ill; 3 = mildly ill; 4 = moderately ill; 5 = markedly ill; 6 = severely ill; 7 = among the most extremely ill patients.^25^

### Statistical analyses

Parametric variables are presented as mean ±  standard deviation, while non-parametric variables are presented as median (interquartile range). Non-parametric comparisons among the three groups were analyzed using the Kruskal–Wallis test. Post hoc analyses were conducted using Dunn's test. Frequency comparisons were performed using either the chi-square test or Fisher's exact test.

The frequency of suicidal thoughts and attempts was compared between the PTSD–MDD and MDD groups using the Pearson chi-square test. The control group was not included in the analysis.

To compare the age of onset of NSSI, analyses were conducted between the PTSD–MDD and MDD groups, excluding nail-biting. The age of onset of nail-biting was compared among the three groups using the Kruskal–Wallis test.

## RESULTS

A total of 153 cases were included in the study, of which 46 were in the PTSD–MDD group, 60 were in the MDD group, and 47 were in the control group. The sociodemographic characteristics, self-harm incidents, suicidal thoughts, and attempts of the subjects were compared across the groups. When sociodemographic characteristics were examined, statistically significant differences were observed in the number of siblings, parents’ educational levels, parents’ marital status, growing in single-parent households, and socio-economic status (*p* < 0.05) (Table 1). The average symptom duration for the PTSD–MDD group was 0.5 ± 0.9 years, while for the MDD group it was found to be 0.3 ± 0.7 years. No significant difference was found between the groups in terms of symptom duration and homogeneity (*p*>0.05).

Individuals in the PTSD–MDD group received diagnoses after incidents of sexual abuse. The data collected included details such as the perpetrator of the sexual abuse, the number of exposure, the age at which the first exposure occurred, and a comprehensive analysis of the specific type of sexual abuse. Among the victims of sexual abuse in childhood and adolescence, 41.3% experienced more than three incidents of abuse. Additionally, three cases (6.5%) became pregnant after the abuse, with two (4.3%) giving birth. Notably, in the PTSD–MDD group, a significant proportion (43.5%) reported incidents of sexual abuse by family members. Incidents involving oral, anal, and vaginal penetration together accounted for 63% of the reported cases. A comprehensive presentation of information on sexual abuse is presented in Table 2.

In the PTSD–MDD group, the prevalence of NSSI was 91.3%, whereas in the MDD group, it was 65%. Significantly higher rates of NSSI prevalence were observed in the PTSD–MDD group, with self-cutting being the most common type at 78.3%. Following self-cutting, the most common NSSI types included self-hitting, scratching, and scab picking. In the MDD group, similar to the PTSD group, self-cutting predominated as the most common NSSI type at 58.3% (Table 3).

In the PTSD–MDD group, 41 (89.1%) reported suicidal ideation, while 24 (52.2%) had attempted suicide. In the MDD group, 33 cases (55%) reported suicidal ideation, with 11 (18.3%) attempting suicide. Both the rates of suicidal ideation and suicide attempts were significantly higher in the PTSD–MDD group. It is noteworthy that children and adolescents who engaged in any form of self-harming behavior were significantly more likely to attempt suicide (Table 3).

Information on bullying, witnessing domestic violence, physical and emotional abuse, and neglect was extracted from previous patient interviews obtained from files. When these data were examined, exposure to emotional and physical abuse, neglect, and domestic violence was higher in the PTSD–MDD group than the MDD group and higher in the MDD group than the control group (*p* < 0.05). Regarding peer bullying rates, the MDD group had the highest rate at 68.1%. This was followed by the PTSD–MDD group at 65.1% and the control group at 12.8%.

When the test results were investigated, the mean CDI scores for individuals in the PTSD–MDD group were 28.8 ± 8.5, whereas for the MDD group, the scores were 27.6 ± 6.8. Analysis of the screen for child anxiety related emotional disorders (SCARED) scores revealed an average score of 41.4 ± 15.0 for the PTSD–MDD group and 39.3 ± 13.1 for the MDD group. No significant differences were found between the groups in terms of anxiety and depression scales. On the clinician-rated CGI scale, the mean score for individuals in the PTSD–MDD group was 5.5 ± 0.7, while in the MDD group, it was 4.6 ± 0.5. The PTSD–MDD group had statistically significantly higher CGI scores than the MDD group. It was observed that the high CDI, SCARED, and CGI results were positively correlated with the rates of suicidal ideation and suicide attempts.

**Figure 1.**
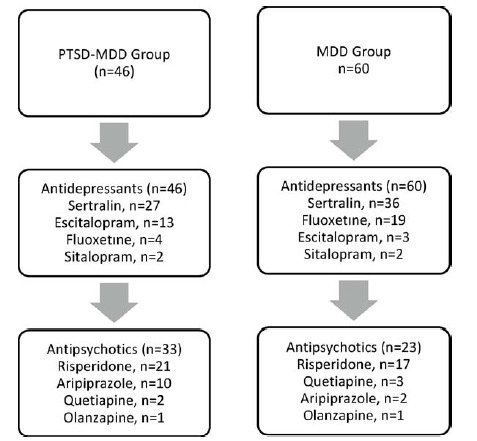
Drug use in the post-traumatic stress disorder (PTSD) and major depressive disorder (MDD) group.

**Figure 2.**
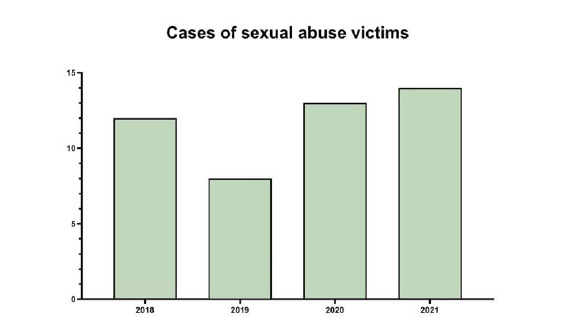
Distribution of clinical presentations of sexual abuse victims by year.

Upon examination of medication use among the case groups, it was clear that there were similar rates of antidepressant use in both the PTSD–MDD and MDD cohorts. Notably, sertraline was the most frequently prescribed medication in both groups. However, when considering antipsychotic medication use, a notable disparity emerged, as individuals in the PTSD–MDD cohort had a significantly higher propensity for antipsychotic drug use compared with those in the MDD group. Risperidone was found to be the predominant antipsychotic agent in both groups. A comprehensive representation of the use of antidepressants and antipsychotics among the cases is shown in Figure 1. The study included 47 sexual abuse victims diagnosed with PTSD–MDD, with 12 presenting in 2018, 8 in 2019, 13 in 2020, and 14 in 2021 (Figure 2).

In the PTSD–MDD group, 52.2% and in the MDD group 18.3% attempted suicide at least once. The rate of three or more lifetime suicide attempts was 17.4% in the PTSD–MDD group and 3.4% in the MDD group. The rates of suicidal thoughts and attempts were significantly higher in the PTSD–MDD group than in the MDD group and significantly higher in the MDD group than in the control group. The frequency of suicide thoughts and attempts in patients with NSSI was examined, and it was found that 92.8% of the patients with NSSI had suicidal thoughts. However, 57.1% of the cases with NSSI attempted suicide.

Predictions of suicide attempt were examined using a logistic regression model. NSSI, history of sexual abuse, CDI score, history of peer bullying, sex, and age were entered into the model using the backward method. NSSI and history of sexual abuse were important variables. The Nagelkerke *R* ^2^ of the model was 0.456. The variables in the model are presented in Table 4. According to these results, NSSI and exposure to childhood sexual abuse increased the risk of a suicide attempt by 12.7-fold and 4.1-fold, respectively.

## DISCUSSION

NSSI is one of the most common psychological problems among children and adolescents.^26^ In our study, we compared 46 cases diagnosed with both PTSD and MDD who were victims of sexual abuse with cases diagnosed with MDD alone (*n* = 60) and a control group (*n* = 47). Through this comparison, we aimed to investigate the relationship between sexual abuse and NSSI and SSI behaviors in children and adolescents. In our study, 89.1% of children and adolescents who were victims of sexual abuse with a diagnosis of comorbid PTSD–MDD thought of suicide, while 52.2% attempted suicide at least once. In the same group, the rate of NSSI was 91.3% (Table 3). In our study, children and adolescents who experienced sexual abuse showed higher levels of NSSI, SSI, and suicide attempts compared with the other groups (Table 3). It was also observed that children and adolescents who self-harmed were four times more likely to attempt suicide than those who did not (Table 4).

The number of cases diagnosed with PTSD–MDD following sexual abuse in the study decreased from 12 in 2018 to 8 in 2019, but subsequently increased to 13 in 2020 and 14 in 2021. Sheila et al. found a relationship between the COVID-19 pandemic and lockdown measures and an increase in the risk of child sexual abuse.^27^ Although the number of cases appeared to decrease in 2019, this could be attributed to the temporary closure of outpatient clinics during the initial phases of the COVID-19 pandemic in Turkey. The increase in numbers in 2020 and 2021 could also be related to the reopening of these clinics.

Many risk factors have been identified in studies of NSSI. One of these risk factors is the diagnosis of PTSD.^26,28,29^ When examining the data in our study, the observed association between the diagnosis of PTSD and suicidal thoughts and attempts is consistent with the findings in the adult literature.^26,28,29^ Our results suggest a notable association between the diagnosis of PTSD and the manifestation of suicidal ideation and attempts. Additionally, we posit that there is a significant correlation between NSSI behavior and the diagnosis of PTSD. Our findings emphasize the need for targeted interventions and heightened vigilance in managing the associated risks, particularly among individuals diagnosed with MDD and PTSD, to reduce the prevalence of self-harming behavior and improve overall mental health outcomes. The literature indicates that psychosocial approaches such as dialectical behavior therapy and emotion regulation therapy are applied to patients with self-injurious behaviors.^30,31^ In addition to these psychosocial interventions, pharmacological treatments, including atypical antipsychotics and serotonin reuptake inhibitors, are also part of the therapeutic approach.^32^ In particular, there is a need for further research into treatment options for adolescent patients who exhibit self-harming behavior.

There were no differences in age and gender among the groups (Table 1). Additionally, there were no statistically significant differences in CDI and SCARED scores between the PTSD–MDD group and the MDD group. Despite similar depression and anxiety scale scores, it was observed that children and adolescents who were victims of sexual abuse were at higher risk of self-harm. This highlights the importance of considering and evaluating sexual abuse in children and adolescents who, despite having similar symptoms of depression and anxiety, exhibit severely impaired functioning, engage in self-harm, and attempt suicide. In a review study by Maniglio et al. when analyzing the results of 177 studies that included both adolescents and adults, childhood sexual abuse was shown to be a risk factor for self-harming behavior.^33^ Furthermore, close monitoring of cases of known sexual abuse in terms of suicidal ideation and attempts is advisable.

The study showed that children and adolescents who were exposed to sexual abuse were more at risk of physical and emotional abuse and neglect than those who were not. Furthermore, 62.5% of children and adolescents who were victims of sexual abuse were neglected and witnessed domestic violence. Physical abuse was observed in 84.8% of them, and emotional abuse was observed in all of them. In a cross-sectional study by Andersson et al. involving 3,169 adolescents, sexual, physical, and emotional abuse were found to be associated with NSSI behaviors.^34^ A relationship between childhood sexual abuse and self-harm behavior was found in studies conducted in countries such as Brazil, the United States, and Sweden, as well as Turkey, where our study was conducted.^28,34,35^ We suggest that children and adolescents can be protected from future abuse and suicide attempts by taking the necessary precautions against any type of abuse. In the study by Barbosa et al., cases aged 14–35 years were included, and all types of abuse (sexual abuse, physical abuse, and emotional abuse) were found to increase NSSI rates.^35^ Barbosa's study also showed that untreated childhood pathologies lead to mental disorders that persist into adulthood.^35^ Lahav's study also showed that 23.8% of adults who were exposed to neglect and abuse during childhood had suicidal thoughts in the past 12 months.^36^

In our study, retrospective screenings delved into cases of exposure to domestic violence, neglect, emotional and physical abuse, as well as cases of peer bullying. The PTSD–MDD and MDD groups were exposed to higher rates of peer bullying than the control group. Unlike other types of abuse and neglect, bullying is more common in the MDD group than in the PTSD–MDD group. The study by Christoffersen et al. showed that peer bullying increased NSSI six times.^29^ We believe that implementing educational interventions focused on bullying has the potential to reduce the rates of bullying among children and adolescents and enhance their coping mechanisms against peer victimization. Considering the findings of our study and the existing literature, it is important to recognize that reducing peer bullying may also prove effective in preventing suicidal and NSSI behaviors. By addressing the root problem of bullying, interventions can play a crucial role in fostering a safer and healthier environment for the well-being of young individuals.

There are many treatment plans for PTSD and MDD psychopathologies.^37^ Many studies have shown that trauma-focused cognitive behavioral therapy (CBT) is beneficial in treating PTSD–MDD cases diagnosed after childhood trauma.^38,39^ When necessary, psychopharmacological treatments are used in addition to CBT. In our analysis of psychometric assessments, we found that although the PTSD–MDD group had similar symptom severity to the MDD group, individuals in the PTSD group used significantly more antipsychotic medications.

Our study has several limitations. The retrospective cross-sectional design prevented us from observing the direct effects of prescribed medical treatments on symptoms. Specifically, we investigated self-harm behaviors and suicide prevalence in a subset diagnosed with PTSD–MDD following sexual abuse. However, due to the retrospective nature of the study, exclusion of patients with incomplete data resulted in a relatively small sample size (46 PTSD–MDD cases, 60 MDD cases, and 47 control cases). This limitation may restrict the generalizability of our findings. Another limitation of our study is the lack of specific information about the duration and timing of exposure to domestic violence, although data were available on whether the cases witnessed domestic violence.

Children diagnosed with PTSD–MDD following sexual abuse showed depressive mood, anhedonia, and feelings of guilt and worthlessness similar to those with MDD. However, significantly higher rates of NSSI and suicide attempts were observed in these children. In future studies with larger sample sizes, comparisons with different trauma etiologies, consideration of family support systems, and evaluation of personal coping skills may contribute to the literature.

## CONCLUSION

The findings of this study highlight an elevated risk of self-harm and suicidal behaviors among individuals diagnosed with PTSD. Children diagnosed with PTSD are at higher risk of both suicidal and non-suicidal self-injurious behaviors than those diagnosed with MDD. Despite similar levels of depressive symptoms as assessed by psychometric tests, individuals who experienced sexual abuse show higher rates of self-injurious behaviors. It is recommended that children and adolescents who exhibit NSSI behaviors such as cutting, hitting themselves, or scratching be subjected to more thorough evaluation for suicidal ideation and attempts. Furthermore, clinicians should consider the possibility of sexual abuse among children and adolescents who engage in self-harm or attempt suicide. As previously discussed, studies in Brazil, the United States, Sweden, and Turkey also reveal an association between childhood sexual abuse and self-harming behavior. It is worth noting that children and adolescents who have attempted suicide often seek help from psychiatric clinics or emergency departments.

Given the frequency of intrafamilial sexual abuse documented in the study, it is suggested that children and adolescents who exhibit suicidal ideation or engage in NSSI behavior may benefit significantly from careful care by emergency department physicians and subsequent referral to psychiatric care.

### Data availability statement

The SPSS data used to support the findings of this study are restricted by the Ethics Committee of Yenimahalle Education and Research Hospital at the University of Ankara Yildirim Beyazit to protect patient privacy. Data are available from Helin Aburşu for researchers who meet the criteria for access to confidential data.

### Competing interests

The authors have no conflicts of interest to declare.

## Figures and Tables

**Table 1:** Demographic characteristics of patients and parents.

Demographic characteristics	PTSD–MDD (*N* = 46)	MDD (*N* = 60)	Control (*N* = 47)	χ^2^ or *Z*	*p*

Age, mean (min–max)	15.5 (8–17)	15.0 (9–17)	15.0 (8–17)	3.344	0.188

Sex, *N* (%)					

Male	6 (13)	11 (18.3)	8 (17.0)	0.556	0.757

Female	40 (87)	49 (81.7)	39 (83.0)		

Number of siblings, mean (min–max)	1.5 (0–6)^a^	1.0 (0–5)^b^	2 (0–5)^a^	11.445	0.003

Demographic characteristics of parents					

Mothers’ age, mean (min–max)	41.5 (33–55)	40 (32–55)	40 (32–55)	2.006	0.367

Fathers)’ age, mean (min–max)	45 (35–55)	45 (35–65)	45 (35–65)	1.850	0.396

Literality of mothers, *N* (%)					

Primary and secondary	34 (73.9)	31 (51.7)	15 (31.9)	16.452	< 0.001

High school and college	12 (26.1)	29 (48.3)	32 (68.1)		

Literality of fathers, *N* (%)					

Primary and secondary	28 (60.9)	22 (36.7)	7 (14.9)	21.036	< 0.001

High school and college	18 (39.1)	38 (63.3)	40 (85.1)		

Mothers’ occupation					

Employed, *N* (%)	18 (39.1)	24 (40)	16 (34.0)	2.912	0.573

Unemployed, *N* (%)	28 (60.9)	36 (60)	31 (65.9)		

Fathers’ occupation					

Employed, *N* (%)	40 (87)	58 (96.7)	45 (95.7)	4.186	0.123

Unemployed, *N* (%)	6 (13.1)	2 (3.4)	2 (4.3)		

Parents’ marital status					

Married, *N* (%)	22 (47.8)	50 (83.3)	42 (89.4)	26.115	< 0.001

Divorced, *N* (%)	22 (47.8)	8 (13.3)	5 (10.6)		

Widowed, *N* (%)	2 (4.3)	2 (3.3)	0 (0.0)		

Growing with a single parent, *N* (%)	20 (43.5)	5 (8.3)	6 (12.8)	22.265	< 0.001

Living in, *N* (%)					

Province	4 (8.7)	2 (3.3)	2 (4.3)	3.060	0.548

Town	42 (91.3)	58 (96.7)	45 (95.7)		

Socio-economic status					

Low, *N* (%)	12 (26.1)	2 (3.3)	4 (8.5)	29.630	< 0.001

Middle, *N* (%)	30 (65.2)	40 (66.7)	19 (40.4)		

High, *N* (%)	4 (8.7)	18 (30)	24 (51.1)		


Similar superscript letters indicate no significant difference between the groups. Statistically different groups are shown with different superscript letters. The *p* values in bold are significantly different at *p* < 0.05. PTSD: post-traumatic stress disorder, MDD: major depressive disorder, N: number, min: minimum, max: maximum.

**Table 2:** Characteristics of sexual abuse victims and perpetrators.

Characteristics of sexual abuse	PTSD–MDD, *N* = 46 (%)

Age of onset, mean (min–max)	13 (4–17)

Number of reported sexual abuse	

Once	16 (34.8)

Twice	9 (19.6)

Three times	2 (4.3)

Four or more	19 (41.3)

Identity of perpetrator	

Boyfriend	8 (17.4)

Friend	6 (13.0)

Acquaintance	6 (13.0)

Family member	20 (43.5)

Stranger	6 (13)

Penetration	29 (63)

Pregnancy	3 (6.5)

Curettage	1 (2.2)

Giving birth	2 (4.3)


PTSD: post-traumatic stress disorder, MDD: major depressive disorder, N: number, min: minimum, max: maximum.

**Table 3:** Non-suicidal self-injury (NSSI) characteristics.

NSSI characteristics	PTSD–MDD *N* = 46 (%)	MDD *N* = 60 (%)	Control *N* = 47 (%)	χ^2^	*p*

Prevalence of NSSI	42 (91.3)^a^	39 (65)^b^	10 (21)^c^	48.549	< 0.001

Type of self-injury					

Self-cutting	36 (78.3)^a^	35 (58.3)^b^	0 (0.0)^c^	59.525	< 0.001

Self-hitting	30 (65.2)^a^	8 (13.3)^b^	0 (0.0)^c^	59.963	< 0.001

Banging head	5 (10.9)^a^	2 (3.3)^b^	0 (0.0)^b^	8.431	0.070

Cuticle biting	21 (45.7)^a^	12 (20)^b^	4 (8.5)^b^	18.433	< 0.001

Burning	0 (0.0)^a^	1 (1.7)^a^	0 (0.0)^a^	1.882	0.390

Punching	20 (43.5)^a^	11 (18.3)^b^	1 (2.1)^c^	24.429	< 0.001

Pinching	11 (23.9)^a^	10 (16.7)^a^	0 (0.0)^b^	11.947	0.003

Pulling hair	16 (34.8)^a^	8 (13.3)^b^	0 (0.0)^c^	21.679	< 0.001

Scab picking	33 (71.7)^a^	10 (16.7)^b^	0 (0.0)^c^	65.603	< 0.001

Nail-biting	5 (10.9)^a^	4 (6.7)^a^	7 (14.9)^a^	1.936	0.380

Scratching	32 (69.6)^a^	14 (23.3)^b^	2 (4.3)^c^	49.019	< 0.001

Suicidal thoughts	41 (89.1)	33 (55.0)	0 (0.0)	14.392	< 0.001

In patients with NSSI	39 (95.1)	30 (90.9)	0 (0.0)	0.512	0.474

In patients without NSSI	2 (4.9)	3 (9.1)	0 (0.0)		

	χ^2^ = 4.468 p = 0.035	χ^2^ = 21.638 p < 0.001			

Suicide attempts	24 (52.2)	11 (18.3)	0 (0.0)	13.482	< 0.001

In patients with NSSI	24 (100)	10 (90.9)		2.380	0.123

In patients without NSSI	0.0 (0.0)	1 (9.1)			

	χ^2^ = 6.318 p = 0.012	χ^2^ = 4.726 p = 0.030			


Similar superscript letters indicate no significant difference between the groups. Statistically different groups are shown with different superscript letters. The *p* values in bold are significantly different at *p* < 0.05. PTSD: post-traumatic stress disorder, MDD: major depressive disorder, N: number.

**Table 4:** Logistic regression model analysis of suicide attempt predictions.

	B	OR	*p*	95% CI

Constant	-3.744	0.024	< 0.001	

NSSI	2.545	12.745	0.017	1.579–102.843

Sexual abuse	1.417	4.125	0.003	1.625–10.471

CDI score	0.070	1.073	0.009	1.018–1.131


B: unstandardized coefficient, OR: odds ratio, CI: confidence interval, Constant: predicted value of the dependent variable when all independent variables are zero, NSSI: non-suicidal self-injury, CDI: child depression inventory, Nagelkerke *R* ^2^: 0.456.
